# Recent Advances in Self-Powered Tactile Sensing for Wearable Electronics

**DOI:** 10.3390/ma17112493

**Published:** 2024-05-22

**Authors:** Ling-Feng Liu, Tong Li, Qin-Teng Lai, Guowu Tang, Qi-Jun Sun

**Affiliations:** School of Physics and Optoelectronic Engineering, Guangdong University of Technology, Guangzhou 510006, Chinaguowutang@126.com (G.T.)

**Keywords:** self-powered tactile sensors, high sensitivity, health monitoring, artificial intelligence

## Abstract

With the arrival of the Internet of Things era, the demand for tactile sensors continues to grow. However, traditional sensors mostly require an external power supply to meet real-time monitoring, which brings many drawbacks such as short service life, environmental pollution, and difficulty in replacement, which greatly limits their practical applications. Therefore, the development of a passive self-power supply of tactile sensors has become a research hotspot in academia and the industry. In this review, the development of self-powered tactile sensors in the past several years is introduced and discussed. First, the sensing principle of self-powered tactile sensors is introduced. After that, the main performance parameters of the tactile sensors are briefly discussed. Finally, the potential application prospects of the tactile sensors are discussed in detail.

## 1. Introduction

Among human senses, touch is one of the most significant. It can provide us with important information about the physical world around us, such as shape, size, temperature, and so on [1]. In the Information Era, data acquisition and processing are inseparable from the participation of sensors. They are widely used in robotics [2,3,4,5], motion monitoring [6,7,8,9], medical, human–machine interaction [10], and other fields [11,12,13]. However, traditional tactile sensors rely on external power sources for their power supply, which can lead to problems such as large system size, high cost, and difficult maintenance. These shortcomings limit their use in some scenarios. The self-powered tactile sensor, however, does not require external power support, and can use environmental energy to achieve self-power supply [14,15], thus solving the defects of traditional tactile sensors.

Self-powered tactile sensors are more flexible, weigh less, and have a lower volume than conventional pressure sensors. At the same time, they have higher pressure resolution, spatial resolution, and sensitivity. The self-powered tactile sensor can gather energy from the surrounding environment such as mechanical energy [16], thermal energy [17], wave energy [18], etc., and convert this energy into electrical power to achieve an autonomous power supply. The characteristics of working without relying on external batteries have greatly broadened its application possibilities, such as in aerospace, marine engineering, unmanned submersibles, oil and gas drilling, and underground mining.

Despite the fact that many academics have made great progress in the study and use of self-powered touch sensors, some issues still need to be addressed. First, the efficiency and reliability of self-powered technology should be further improved to meet the needs of the sensor. Second, the sensitivity, accuracy, and stability of the self-powered tactile sensor should be further improved to meet the needs of different application scenarios. In addition, since the self-powered tactile sensor needs to work in a complex environment, its anti-interference and reliability also need to be further optimized. Finally, the manufacturing process of self-powered tactile sensors also needs to be improved to satisfy the needs of large-scale applications. Self-powered tactile sensors have lots of potential and merit further exploration.

Many academics are researching the passive self-power supply of tactile sensors. By consulting studies from all over the world, this paper reviews the sensing principle and main performance indexes of self-powered tactile sensors, and puts forward their potential applications and issues.

## 2. Classification of Self-Powered Tactile Sensors

**Figure 1:**
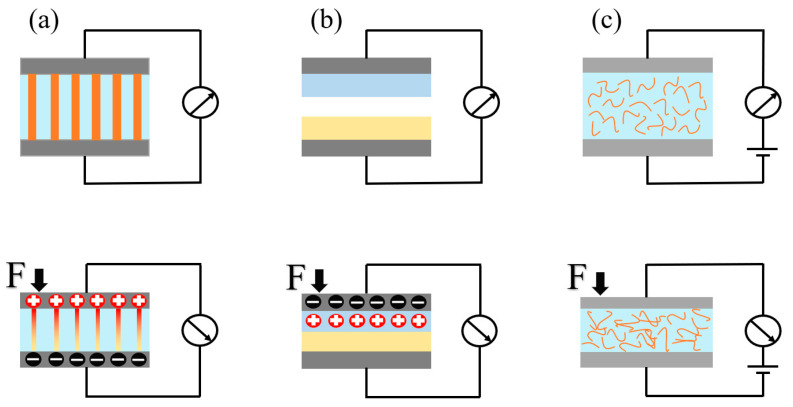
The sensing mechanisms of the typical tactile sensor. (**a**) Piezoelectric-type. (**b**) Triboelectric-type. (**c**) Piezoresistive-type.

Commonly used self-powered tactile sensors can be categorized into three types based on their sensing mechanism: piezoelectric, triboelectric types, and piezoresistive. The sensing mechanisms of the typical self-powered tactile sensor are depicted in Figure 1. In the following sections, the fabrication techniques, working principles, and applications of these three types of self-powered tactile sensors will be introduced and discussed. It should be noted that the piezoresistive tactile sensors have rarely been discussed in previous studies. The self-powered piezoresistive tactile sensors are based on the flexible battery and piezoresistive tactile sensor hybrid sensing systems.

### 2.1. Piezoelectric-Type

The sensing principle of the piezoelectric self-powered tactile sensor (Figure 1a) is mainly based on the positive piezoelectric effect of piezoelectric materials [19,20]. When an external force is applied to the piezoelectric material, polarization occurs inside the active layer. The positive and negative charge centers inside the material shift, and then opposite polarity and equal magnitude charges appear on the upper and lower relative surfaces, forming an electric field inside the material. When the external force is removed, the piezoelectric material will slowly recover to an undeformed state, and the charged state will disappear [21].

**Figure 2:**
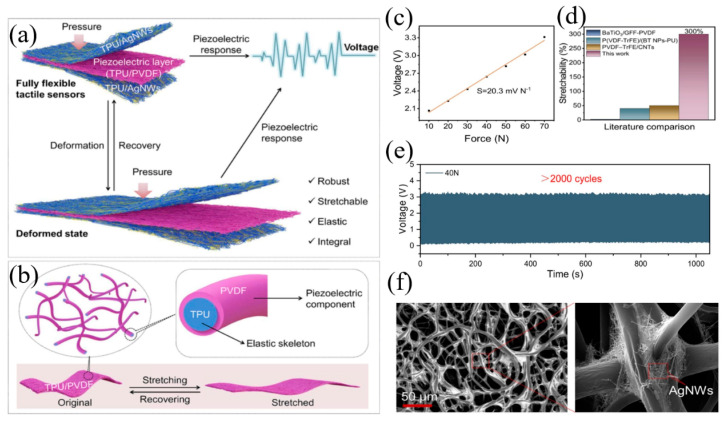
The fully flexible tactile sensor. Reproduced from [37] with permission from Elsevier, copyright 2023. (**a**) The structure of the sensor and its piezoelectric response. (**b**) The structure of a core–shell structured fibrous piezoelectric mat. (**c**) Output voltage under different forces and the linear fitting curve. (**d**) Comparison of the flexibility of the PVDF-based tactile sensors with the fully flexible tactile sensor in this study. (**e**) Tactile sensor’s piezoelectric reaction to a cyclic force over 2000 cycles. (**f**) The surface morphology of a TPU/AgNW electrode.

Piezoelectric constant d_33_ is the main parameter to describe the properties of piezoelectric materials, which illustrates their capacity to transform mechanical energy into electrical energy. The larger the d_33_, the better the piezoelectric properties of piezoelectric materials [22]. Based on their chemical makeup, the widely used piezoelectric materials can be divided into three categories: inorganic, organic, and composite. Inorganic piezoelectric materials generally use PZT, BaTiO_3_, PbTiO_3_, etc. [23,24,25]. Inorganic piezoelectric materials such as PZT have high piezoelectric parameters, but these inorganic materials have the disadvantage of excessive brittleness, which limits their development in the field of flexibility [26]. In addition to being used as a dopant to create polymer piezoelectric ceramic composites [27,28,29], researchers have worked hard to address the drawbacks of inorganic piezoelectric materials in an effort to make significant progress. AlN is a popular choice among piezoelectric materials due to its high signal-to-noise ratio, low dielectric loss tangent, high temperature stability, and excellent piezoelectric and dielectric characteristics [30,31]. Commonly used organic materials are polyvinylidene fluoride [32,33,34] (PVDF) and polydimethylsiloxane [35,36] (PDMS). Among them, PVDF has good flexibility, chemical stability, and excellent electromechanical conversion, which makes it a material with a lot of potential applications in the tactile sensor industry. In order to enhance the function of the piezoelectric tactile sensor, scientists have tried various techniques. These methods include refining the composition, structure, or preparation method of piezoelectric materials, optimizing the structural design of the sensor, including its size, shape, and electrode arrangement, and combining the piezoelectric tactile sensor with other sensor types (such as optics, capacitance, and so on) to complete multimodal data collecting and in-depth analysis. Wei et al. prepared a fully flexible and mechanically robust sandwich-like tactile sensor by introducing a core-shell fiber piezoelectric pad between two fibrous TPU/Ag NWs flexible electrodes (Figure 2) [37]. The sensitive layer of the sensor was prepared by coaxial electrospinning, which was composed of TPU and PVDF fiber piezoelectric felt with core-shell structure. The elastic skeleton in the core is composed of TPU, which is used to reinforce the mechanical properties, and PVDF is directly wrapped on the TPU fiber as a piezoelectric element. The piezoelectric felt with core-shell structure exhibits superior piezoelectric properties, and has better elasticity and stretchability. Because both the sensitive layer and the electrode have excellent flexible properties, the elongation of the prepared tactile sensor reaches 300%, and the outstanding elasticity is superior to most PVDF-based piezoelectric materials reported in the past. The sensitivity of the sensor is 20.3 mVN ^−1^, and it is able to sense more than 2000 cycles stably.

### 2.2. Triboelectric-Type

The sensing principle of triboelectric tactile sensors (Figure 1b) is mainly based on the triboelectric effect [38], which is generally composed of an electrode and two triboelectric materials. When there is an external force, these friction materials make contact with each other. Due to their different abilities in attracting electrons, materials with weak abilities to attract electrons lose electrons with a positive charge, while the others will be negatively charged. After removing the external force, the friction layers will separate from each other. Because of the electrostatic induction, the other side of each friction layer will induce the opposite charge to form the induced potential, so as to realize the tactile perception of the external object.

The triboelectric (TENG) tactile sensor has the advantages of low cost, simple device construction, high output voltage, and wide material selection. However, the pressure sensitivity of the triboelectric tactile sensors is usually low, which limits their practical applications [39,40]. Numerous approaches have been attempted by researchers to improve the sensitivity of TENG-based tactile sensors, such as employing materials (carbon nanotubes and polymer composites, etc.) with good mechanical flexibility and friction surface as the friction layers. Preparing micron or nanometer structures on the surface of the friction layers has been demonstrated as one of the most effective approaches to improve the sensitivity of TENG-based tactile sensors [41,42,43,44]. The main preparation methods of microstructures are lithography, 3D printing, and molding, which can only generate fixed microstructures and are costly [45,46,47,48]. In order to change the sensitivity, people can only reconstruct, which is very energy-consuming and expensive.

**Figure 3:**
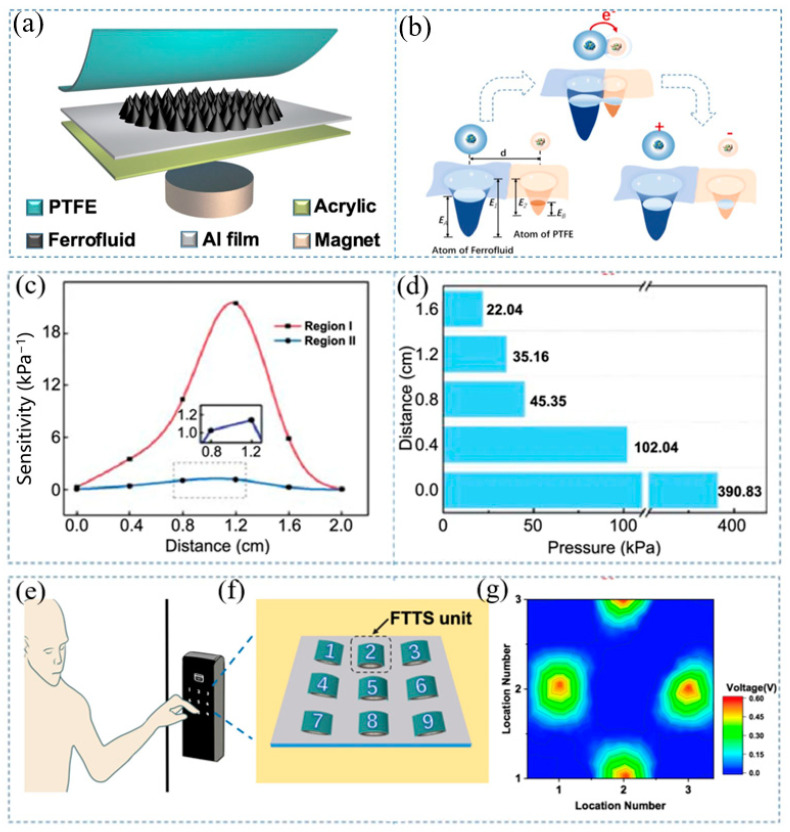
Triboelectric tactile sensor based on ferromagnetic fluid. Reproduced from [49] with permission from Springer, copyright 2022. (**a**) Basic structure diagram. (**b**) The principle of contact electrification. The variation of (**c**) sensitivity and (**d**) detection range caused by the change of the distance between the sensor and the magnet. (**e**) A person keying in a passcode. (**f**) The sensor is applied to a nine-bit password lock. (**g**) Under the same external pressure, the password is “2468” voltage contour map.

Liu et al. employed ferromagnetic fluid as friction material to create a friction electric tactile sensor with ultra-high sensitivity (Figure 3) [49]. By changing the position of the external magnet, the morphology of the microstructure can be flexibly adjusted. The sensitivity of this triboelectric sensor reaches 21.48 kPa^−1^, while its pressure detection range reaches 390 kPa. The sensor has demonstrated potential in smart homes and the Internet of Things.

The future development direction of triboelectric tactile sensors could be to integrate more functions such as temperature, humidity, and other sensor functions to adapt to complex environments, and provide more comprehensive perception by continuously improving sensitivity, accuracy, and stability. 

### 2.3. Thermoelectric Effect Type

**Figure 4:**
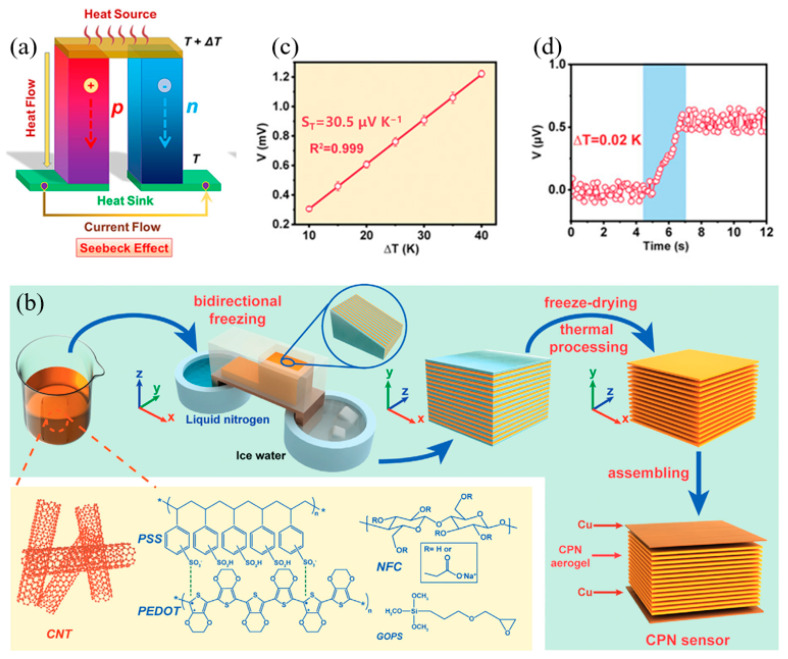
The Seebeck effect and non-contact temperature sensor using the Seebeck effect. (**a**) The principle of the Seebeck effect. Reproduced from [50] with permission from Elsevier, copyright 2023. (**b**) The schematic diagram of sensor preparation. (**c**) The output voltage at different temperatures. (**d**) Voltage output at a small temperature difference of 0.02 K. Reproduced from [55] with permission from Wiley, copyright 2023.

The sensing principle of a thermoelectric tactile sensor (Figure 4a) is mainly based on thermoelectric effect [50]. The thermoelectric effect refers to the electrons in the object moving from the hotter region to the colder region due to the temperature difference, thereby generating current [50]. Thermal energy is one of the most commonly used types of clean energy, but thermoelectric tactile sensors have not been widely used thus far. The main reason is that the thermoelectric energy conversion efficiency of the material is relatively low. ZT is a parameter that characterizes the performance of thermoelectric materials [51]. Generally speaking, thermoelectric performance improves with a greater ZT value [52].

Owing to its low thermal conductivity, theoretically projected high Seebeck coefficient, and ZT, MoS_2_ is regarded as a promising thermoelectric material among two-dimensional nanomaterials [53]. But at the same time, its large direct band gap of 1.2–1.9 eV makes it insulating. As a gapless semimetal, graphene has higher conductivity than MoS_2_. Therefore, the researchers prepared nanocomposites of the above two materials and integrated their advantages. Based on the thermoelectric effect, Xie et al. reported a flexible thermoelectric nanogenerator based on molybdenum disulfide/graphene nanocomposites, which can also be used as a self-powered sensor to detect temperature [54]. Graphene sensors have gained a lot of attention due to their high sensitivity, wide detection range, quick response time, mechanical toughness, and long-term stability. However, graphene synthesis is extremely difficult, limiting the large-scale production of graphene sensors. In recent years, the discovery of laser-induced graphene (LIG) has opened up new possibilities for the low-cost and large-scale manufacturing of high-performance physical sensors. Numerous high-performance sensors and energy devices based on LIG have been described thus far.

Gao et al. designed a temperature sensor with high resolution based on carbon nanotubes/PEDOT: PSS/nanocellulose layered thermoelectric gel (Figure 4) [55]. The sensitivity of this is 30.5 μV K^−1^ and the pressure detection range is as low as 0.02 K. It has great stability and fast temperature response, and it is not interfered with by external pressure. It shows excellent potential in non-contact extraction, decoding, and information transmission applications. The thermoelectric self-powered tactile sensor has attracted much attention because it is environmentally friendly [56,57]. In future, the efficiency of thermoelectric devices is expected to improve to further enhance their application in self-powered pressure and temperature sensing.

### 2.4. Piezoresistive-Type

The sensing principle of piezoresistive tactile sensors is mainly based on piezoresistive effect. When there is an external force, the internal material will deform and cause the resistance value to change. The piezoresistive tactile sensor has attracted much attention due to its advantages of high sensitivity, wide detection pressure range, simple structure, and easy integration [58,59]. However, the traditional piezoresistive tactile sensor needs an external power supply to convert the change of the resistance value into a current or voltage signal (Figure 1c), which greatly limits its application scenarios. 

**Figure 5:**
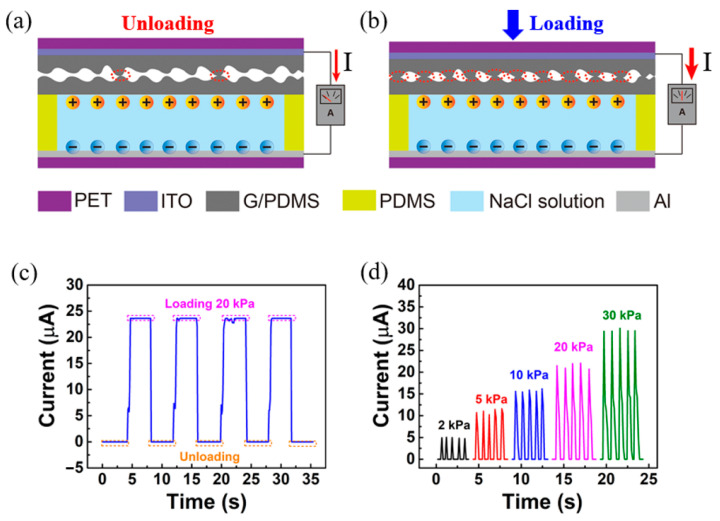
The sensing principle of a self-powered voltage resistive sensor based on redox reaction. Reproduced from [61] with permission from ACS, copyright 2020. (**a**) Not under pressure. (**b**) Under pressure. (**c**) The static response at a pressure of 20 kPa. (**d**) The dynamic response at a pressure of 2, 5, 10, 20, 30 kPa, respectively.

Most researchers achieve the purpose of self-powered sensors by integrating energy harvesters. For example, by using an integrated piezoelectric energy harvester, Yu et al. developed the self-powered piezoelectric tactile sensor [60]. In order to achieve self-power supply, it can also employ thermoelectric or photoelectric materials. While the latter creates electrical energy by altering the optical characteristics of the photoelectric material while the sensor is operating, the former turns heat energy into electrical energy through the tiny amount of friction or deformation caused by the sensor while it is operating. In contrast, Sun et al. designed a self-powered voltage-resistive pressure sensor by using the redox reaction between Al electrode and graphite/PDMS composite (GPC) electrode [61]. The redox potential between the anode and the cathode is the only factor that affects the open circuit voltage in the sensing system. Under the external force, the circuit resistance value changes, and the current signal caused by pressure change can be measured without an external power supply. Its sensing mechanism is shown in Figure 5.

In light of the technical challenges associated with energy harvesting, storage, and management, there is no doubt that the design of piezoresistive tactile sensors is more complex, resulting in increased research costs and time. The trend to move from piezoresistive tactile sensors to self-powered is partly because they are more environmentally friendly and more portable than tactile sensors requiring an external power supply [62,63,64].

## 3. Main Performance Indicators

### 3.1. Sensitivity

Sensitivity is one of the important indexes that estimate the performance of the sensor. It refers to the sensitivity of the sensor’s output response to the change of the measured physical quantity. The accuracy and stability of the measurement can be intuitively reflected by sensitivity. The development of high sensitivity is an area that has been researched thoroughly because high sensitivity is the key to detecting weak signals. The sensitivity of the tactile sensor can be expressed as $S=\delta \left(∆X/X_{0}\right)/\delta P$. *X*_0_ represents the value of the initial electrical signal of the sensor, such as current, voltage, etc.; ∆*X* represents the relative variation of the electrical signal. Generally, the sensitivity can be improved by reducing the intensity of the initial electrical signal. The commonly used methods include the introduction of microstructure [65,66,67], the use of new sensing materials [68,69,70], the introduction of small cracks [71,72,73], etc. The addition of bionic cracks has a number of benefits. First, it can enhance the ability of the sensor to sense changes in external pressure by creating a tiny concave-convex structure on its surface. Additionally, it can mimic the texture of animal or human skin in order to mimic the structure of biological detecting organs, bringing the sensor closer to the mode of biological sensing organs. But at the same time, the introduction of cracks will lead to stress concentration, which will reduce the fatigue life of the material. Therefore, developing strong and damage-resistant materials by resisting the generation and growth of cracks has become the focus of researchers.

**Figure 6:**
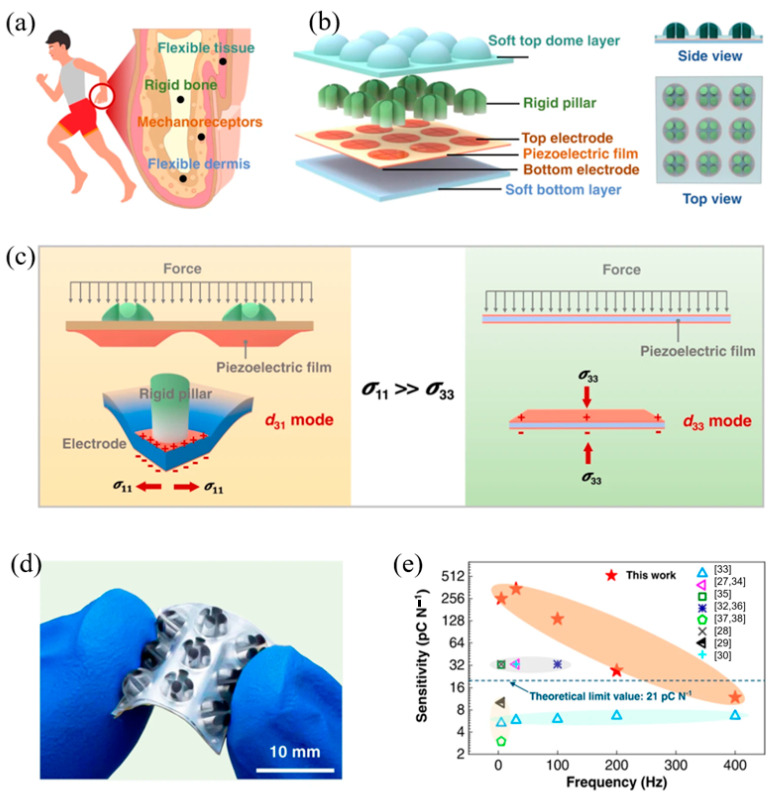
The rigid-soft hybrid tactile sensor inspired by the structure of a finger. Reproduced from [74] with permission from Springer, copyright 2022. (**a**) The internal structure of a finger. (**b**) The internal structure of the sensor and the sensor array. (**c**) The comparison between this sensor and the traditional piezoelectric tactile sensor in the deformation and working mode of the piezoelectric film. Blue indicates the stress distribution of the piezoelectric film. The blue color indicates the stress distribution of piezoelectric film. (**d**) Photograph of a 3 × 3 RSHTS array. (**e**) The difference in sensitivity between this sensor and other piezoelectric tactile sensors (The reference numbers in the figure are those in reference [74]).

Inspired by the structure of skin collagen, Jin et al. proposed an ultra-stable bionic crack sensor, which uses silver nanowire grids as crack arrest layers to control crack depth [2]. The sensor detected a GF of 2000 at 0.5% strain. When integrated into a small-legged robot, the sensor enables the robot to accurately identify the terrain and its current state. Additionally, inspired by the structure of human fingers, Zhang et al. reported a piezoelectric tactile sensor using a rigid-soft hybrid force-transmission-layer in combination with a soft bottom substrate (Figure 6) [74]. The sensor overcomes the dynamic sensitivity limitation of the traditional piezoelectric flexible tactile sensor. The sensitivity is as high as 346.5 pCN^−1^, and it can reliably identify forces in multiple directions, showing much potential in the field of robotics.

### 3.2. Pressure Detection Range

The tactile system of the human body not only has extremely high sensitivity, but also has a wide detection range, which can perceive a breeze (<1 kPa) and griping (50–600 kPa). The pressure detection range specifies the scope of the scene and the objects to which the sensor can be applied. Aside from high sensitivity, wide pressure detection range is also very important for self-powered tactile sensors. However, it is challenging to reconcile the paradox of great sensitivity with a broad pressure detection range, because a large detection range necessitates superior toughness to prevent mechanical damage, but high sensitivity and low detection limits demand low modulus to respond to minor deformation [75,76,77].

**Figure 7:**
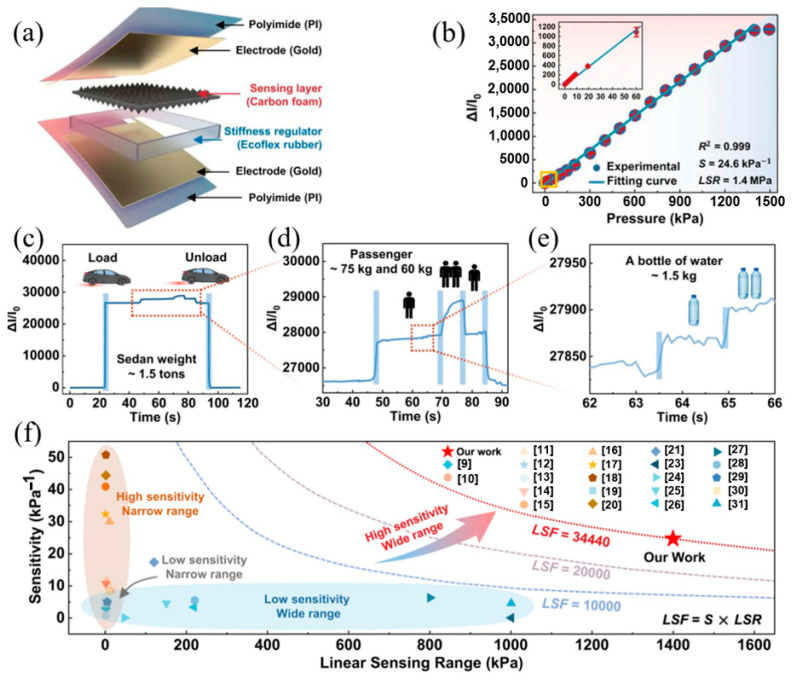
The practical application of the force-electric nonlinear cooperative pressure sensor in the field of physiological monitoring. Reproduced from [78] with permission from Springer, copyright 2023. (**a**) The internal structure design of the sensor. (**b**) The relative current measured from a sensor in the range of 0–1400 kPa. In the figure, S is the sensitivity and LSR is the linear sensing range. (**c**–**e**) The change of relative current caused by the change of load weight of a 1.5 ton car. (**f**) Comparison of performance between this sensor and pressure sensors reported in other literature. LSF represents the linear sensing factor (The reference numbers in the figure are those in reference [78]).

Chen et al. proposed a novel “force-electric” coupling nonlinear collaborative design strategy [78], which breaks through the dilemma of high sensitivity and wide linear range (Figure 7). In order to detect a sensitivity and broad linear range at the same time, they designed a double-sided pyramid porous conductive microstructure and a mechanical regulator as a pressure sensitive layer and a pressure buffer layer, respectively, and then stacked and encapsulated them into the overall structure of the sensor. At the same time, the high-temperature pyrolysis process was used to obtain the conductivity of the pyramid porous structure, which further reduced the size of the micro-pyramid structure. Both the sensitivity (24.6 kPa^−1^) and the linear range (1.4 MPa) of the sensor are excellent, and its linear influence factor (sensitivity range) is much higher than most piezoresistive pressure sensors reported so far.

Zhou et al. proposed a new type of triboelectric pressure sensor, which adopts a novel structure of brushed semi-cylindricity [79]. The semi-prism shape can apparently broaden the detection range, while the brush-like surface structure not only improves the sensitivity, but also reduces the detection limit. Finally, the pressure sensor achieves an ultra-wide detection range (0.04–1200 kPa) and high sensitivity (1305 mV kPa^−1^). It performs better than most of the reported pressure sensors.

### 3.3. Pressure Resolution

The pressure resolution is a very important parameter of the sensor, but is often ignored. It reflects the ability of the tactile sensor to detect small changes in pressure. The higher the pressure resolution, the more accurate the output data [45]. There is still a lack of research on wide-range sensors with high sensitivity and high resolution.

**Figure 8:**
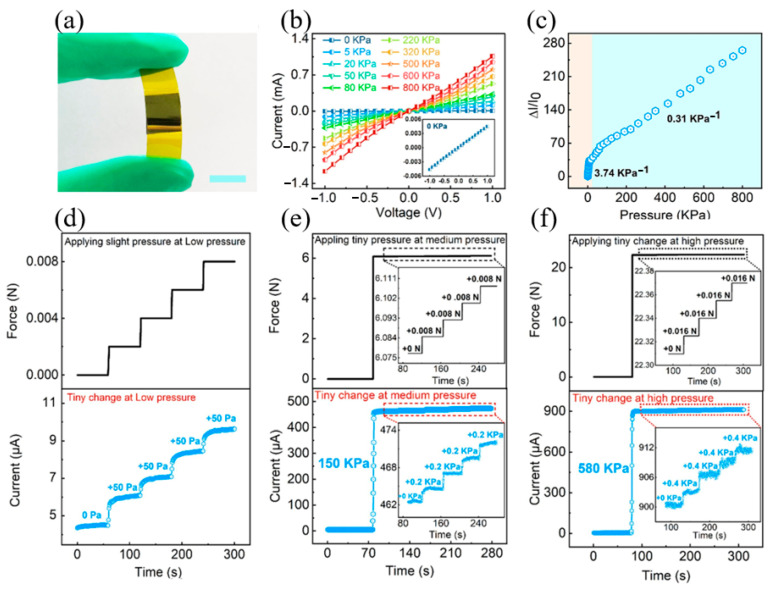
The tactile sensor with gradient pore structure inspired by human skin. Reproduced from [80] with permission from Elsevier, copyright 2022. (**a**) Photograph of the sensor. (**b**) Current-voltage curve measured from one sensor in the range of 0–800 kPa. (**c**) The point diagram of the relationship between ΔI/I0 and pressure. Through the sensitivity calculation formula, the sensitivity within 10 kPa is 3.74 kPa^−^^1^, and the sensitivity between 10 kPa and 800 kPa is 0.31 kPa^−^^1^. (**d**–**f**) When the reference pressure is 0 kPa, 150 kPa and 580 kPa, the resolution is approximately 50 Pa, 200 Pa and 400 Pa, respectively.

Inspired by the structure of human skin, Wang et al. proposed a film based on polydimethylsiloxane with a gradient pore structure to achieve a gradient elastic modulus from top to bottom (Figure 8) [80]. The compressibility and pressure response range of the structure are dramatically improved. The tactile sensor can respond sensitively to a wide pressure range of 0–800 kPa. Its sensitivity is as high as 3.74 kPa^−1^, and the ultrahigh pressure resolution is 0.06%. Its remarkable abilities in medical detection and human-computer interaction demonstrate its enormous promise in the artificial intelligence space.

The performance indexes of most sensors mentioned in this paper are shown in Table 1. In addition to the above three indicators, spatial resolution [81,82], stability [83,84], durability [85,86], flexibility [87,88], and anti-interference ability [89] also play a crucial role in the practical application of sensors.

## 4. Practical Applications

In the area of wearable tech, the self-powered tactile sensor offers a more practical and environmentally friendly option. It can produce electricity using body movement or ambient energy and does not require an external battery module for power supply. This reduces the requirement for battery charge and enhances the wearability and comfort of wearable technology. Because there is no need to replace the battery frequently, maintenance costs are lower and the environmental impact of battery waste is reduced. Wearable sensors are smart devices that are attached to bodies, clothes, or accessories [90]. These sensors can be used for a variety of purposes, including motion tracking, sleep analysis, and health monitoring. Self-powered tactile sensors have a variety of uses in wearable tech.

### 4.1. Human Motion Detection

According to domestic statistics, the incidence of rheumatoid arthritis in China is 0.32–0.42%, the total number of patients is about 5 million, and the disability rate within 5–10 years is as high as 43.48%. Various clinical practice guidelines regard physical activity as a key component in the treatment of arthritis patients. There is also evidence that self-monitoring can effectively improve health behavior. Setting specific goals and reviewing the process can effectively help patients change their physical activity habits and improve their quality of life. Therefore, motion monitoring is important. Self-powered tactile sensors are portable and sustainable, and can convert different movements into electrical signals of different modes or sequences. They are widely used in motion monitoring [91,92,93].

**Figure 9:**
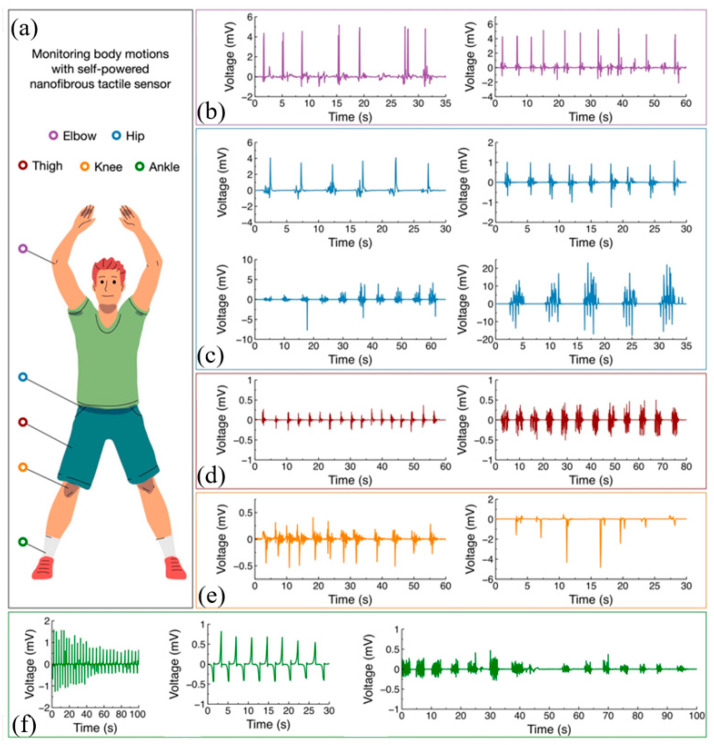
Use PVDF-based self-powered tactile sensors to monitor the movement of various parts of the body. Reproduced from [94] with permission from Springer, copyright 2023. (**a**) The location where the sensor is attached. The waveform generated during periodic activity of various parts of the body. (**b**) Elbow. (**c**) Hips. (**d**) Thighs. (**e**) Knee. (**f**) Ankle.

Li et al. developed a nanofiber membrane based on electrospun polyvinylidene fluoride nanofibers [94]. It is not only self-powered, but also has good flexibility, hydrophobicity, and permeability. As a low-cost and simple tactile sensor, it can be used for human motion detection and recognition. When the sensor is attached to elbows, hips, thighs, knees, and ankles, the generated voltage waveforms can be checked. Each waveform has a unique response (Figure 9). In addition, the tactile sensor has good mechanical and electrical properties, making it well suited for wearable applications.

### 4.2. Health Monitoring

Respiratory rate is an important index by which to judge the health of the human body. Breathing movement can reflect a person’s health status and the prevalence of many respiratory diseases such as sleep apnea syndrome and bronchitis.

Traditional and commercial respiratory monitoring devices have the downsides of difficult portability and high prices, which limit their application in daily life. Since the self-powered breathing sensor based on polyvinylidene fluoride was developed, good results have been achieved with the development of materials science, nanotechnology, and energy harvesting technology. In particular, nanogenerators based on piezoelectric effect and triboelectric effect are promoting the development of self-powered breathing sensors in a smaller, more sensitive, and more accurate direction [95,96,97].

Kim et al. proposed the use of a piezoelectric ZnO nanogenerator based on nylon fabric [98], which has a hierarchical interlocking structure and can also be used as a wearable sensor for biological monitoring. By installing sensors on specific areas of the skin such as the wrist, throat, etc., different physiological activities could be detected, including pulse beats, breathing, and coughing. Additionally, the sensor was fastened to a facial mask, and with the use of a ZnO piezoelectric nanogenerator, they demonstrated high levels of sensitivity in identifying and detecting particular human respiration. The portability of the sensor makes it more convenient to put into daily applications. It can monitor health-related vital signs in real time without affecting the individual’s daily life.

**Figure 10:**
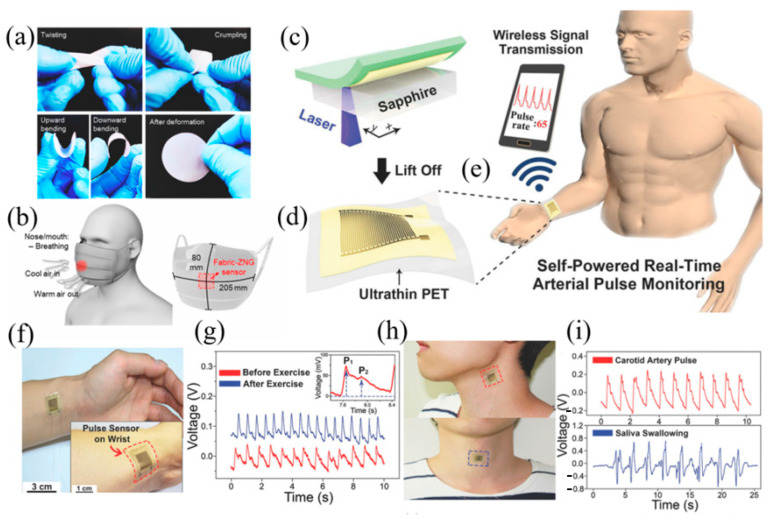
The fabric-based ZnO nanogenerator and the real-time pulse-monitoring system based on PZT thin film. (**a**) Mechanical deformation and resilience of the fabric-ZNG. (**b**) The ZNG device was mounted at the center of the mask to detect the respiration of the nose and mouth. Reproduced from [98] with permission from Elsevier, copyright 2023. (**c**) Peel off PZT thin films deposited on sapphire chips using the ILO process. (**d**) Transfer the peeled PZT film onto a PET substrate. (**e**) The fully fitted wrist sensor transmits the detected pulse signal wirelessly to the smartphone. (**f**) Photo of a sensor fixed to a human wrist using a biocompatible liquid bandage. (**g**) The radial artery pulse signal before and after exercise detected by the sensor. (**h**) Photo of the piezoelectric pulse sensor attached to the carotid artery and the middle of the throat. (**i**) The output voltage in response to carotid artery pressure and saliva-swallowing action. Reproduced from [103] with permission from Wiley, copyright 2017.

Pulse signal is an important indicator for monitoring cardiac status and assessing personal health. In order to achieve long-term continuous pulse monitoring and ensure the accuracy of monitoring results, several research teams have developed portable self-powered pulse sensors with high sensitivity [99,100,101]. Marasco et al. proposed a wearable heart rate monitoring technology based on aluminum nitride (AlN) piezoelectric sensors that can measure heart rate, diastolic, and systolic periods via the posterior tibial artery [102]. Following testing, the system can continually monitor many users at the same time, which is believed to be useful in the medical Internet of Things. Park et al. proposed a self-powered flexible piezoelectric pulse sensor based on PZT thin film [103], which can be used in a real-time medical monitoring system (Figure 10). They used the ILO process to transfer high-quality piezoelectric films onto ultra-thin plastics. The epidermal piezoelectric sensor can be conformally attached to skin to respond to a tiny human pulse. They used MCUs and Bluetooth transmitters to wirelessly transmit pulse signals to smartphones, demonstrating a self-powered real-time pulse-monitoring system.

**Table 1:** The summary of the main performance of the tactile sensor mentioned in this article.

Materials	Sensing Mechanism	Sensitivity	Low Limit	Up Limit	Response Time	Durability [Cycles]	Ref.
TPU/PVDF/ AgNWs	Piezoelectric	20.3 mVN^−1^	NA	NA	70 ms	>2000	[37]
PDMS/PVDF	Piezoelectric	346.5 pCN^−1^	0.009 N	4.3 N	NA	NA	[74]
BTO@PVDF	Piezoelectric	116 mV kPa^−1^ within pressure range 0–10 N	NA	NA	82.7 ms	>12,000	[94]
PZT	Piezoelectric	0.018 kPa^−1^	NA	NA	60 ms	>5000	[103]
PDMS	Triboelectric	1305 mV kPa^−1^	40 Pa	1200 kPa	NA	NA	[79]
Organic-based ferrofluid	Triboelectric	21.48 kPa^−1^	1.25 Pa	390 kPa	90 ms	NA	[49]
graphite/PDMS	Piezoresistive	4.2 × 103 kPa^–1^	20 Pa	30 kPa	8 ms	>4000	[61]
Ecoflex rubber/ melamine foam	Piezoresistive	24.6 kPa^−1^	200 Pa	1.2 MPa	8.4 ms	>5000	[78]
PDMS/CNTs	Piezoresistive	3.74 kPa^−1^	1.65 Pa	800 kPa	15 ms	>5000	[80]
CNT/PEDO:PSS /NFC	Thermoelectric	30.5 μVK^−1^	0.02 K	NA	NA	NA	[55]

### 4.3. Intelligent Robot

Artificial intelligence has entered the phase in which it is subtly changing our lifestyles and quality of life. As the demand for intelligent robots increases, the more difficult it is for them to complete tasks. Tactile sensing technology is crucial for the smart robot; it enables the robot to interact with its environment. To recognize their behavioral state in a more complex environment, the perception capability of robots needs to be further enhanced.

Traditional robots usually use visual and auditory techniques to perceive the external environment. In order to further optimize the application range of robots, inspired by animal whiskers, Liu et al. proposed a bionic whisker sensor with self-powered capability based on triboelectric nanogenerator (TENG) [104]. Its structural design is similar to whiskers, and the inner shell of the follicle is equipped with a touch-sensing device. When a collision occurs, the whisker deviates from the middle position, resulting in an offset between the electrodes, and then an output signal is generated by electrostatic induction.

**Figure 11:**
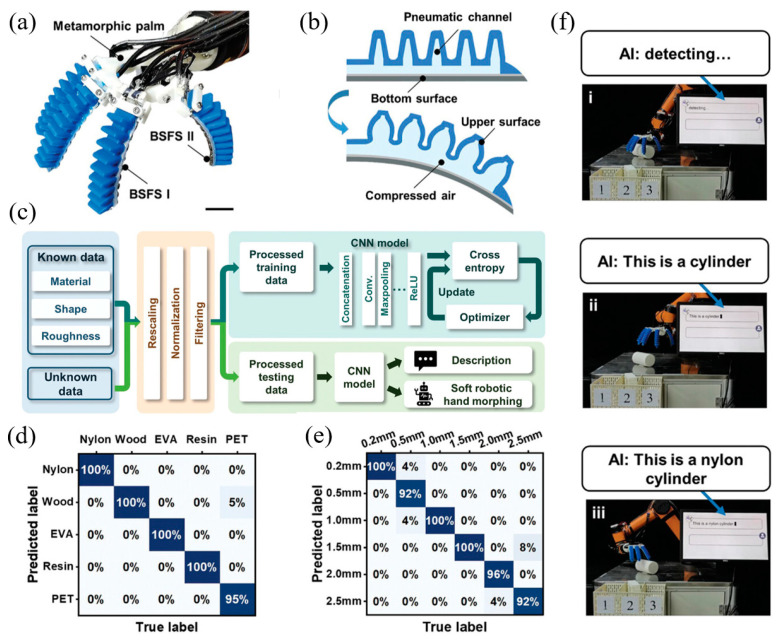
A soft robotic arm with dual-mode and self-powered flexible sensors. Reproduced from [105] with permission from Wiley, copyright 2023. (**a**) Photo of the soft robotic arm. (**b**) The working principle of a mechanical finger. (**c**) Flow chart of perception, description, and classification of objects by an intelligent soft manipulator. (**d**) The confusion matrix for distinguishing different materials (the total accuracy rate reached 99%). (**e**) The confusion matrix for identifying the roughness of the materials (the total accuracy rate reached 97%). (**f**) Photos of an intelligent soft robot system at work.

Because it is difficult for traditional robots that rely on visual and auditory technology to accurately perceive and describe the material properties and surface roughness of objects, researchers are committed to developing intelligent soft robots with flexible multi-modal sensors. Liu et al. proposed a dual-mode self-powered flexible sensor based on a triboelectric nanogenerator [105]. By seamlessly integrating the sensor into its soft finger, they created a humanoid soft manipulator with significant multi-modal sensing capabilities (Figure 11). They also used convolutional neural networks to integrate all sensor information to form an intelligent soft robot system that can accurately describe objects based on their physical characteristics with an accuracy rate of up to 97%.

## 5. Summary and Outlook

In recent years, significant progress has been made in the study of self-powered tactile sensors. This review summarizes the research development of self-powered tactile sensors. It describes the sensing mechanisms and some commonly used materials of piezoelectric, triboelectric, thermoelectric, and piezoresistive tactile sensors. It also introduces the performance indicators of the sensor, such as sensitivity, pressure detection range, pressure resolution, etc. Finally, its practical applications in motion monitoring, pulse and respiratory monitoring, and robotics are discussed.

The emergence of self-powered tactile sensors supports the sustainable development of human society and the improvement of quality of life. The shift of attention from tactile sensor to self-powered tactile sensor is a response to green development. Issues that still need attention are to further improve the performance of self-powered tactile sensors, ensure sensors have better linearity in response to force under different pressures, and more effectively break through the shackles of wide pressure detection range and high sensitivity, and how to integrate multiple sensors on a single device to achieve multi-modal sensing and ensure its functionality and long-term stability. In future, it is hoped that the development of a multifunctional integrated tactile sensor with superior performance, simple fabrication, and low cost, that can serve human society on a large scale, will be realized.

## Data Availability

Not applicable.
